# Total Blood Mercury Levels and Depression among Adults in the United States: National Health and Nutrition Examination Survey 2005–2008

**DOI:** 10.1371/journal.pone.0079339

**Published:** 2013-11-11

**Authors:** Tsz Hin H. Ng, Jana M. Mossey, Brian K. Lee

**Affiliations:** Department of Epidemiology and Biostatistics, Drexel University School of Public Health, Philadelphia, Pennsylvania, United States of America; University of Oxford, United Kingdom

## Abstract

**Background:**

Mercury is a neurotoxicant linked with psychiatric symptoms at high levels of exposure. However, it is unclear whether an association is present at the low exposure levels in the US adult population.

**Materials and Methods:**

Cross-sectional associations of total blood mercury and depression were assessed in 6,911 adults age ≥20 in the National Health and Nutrition Examination Survey (NHANES), 2005–2008. The Patient Health Questionnaire-9 was used to assess depression (high likelihood of a depressive spectrum disorder diagnosis; score 5–27).

**Results:**

Unadjusted survey weighted logistic regression suggested that higher total blood mercury was associated with lower odds of depression (Odds Ratio  = 0.49, 95% Confidence Interval: 0.36–0.65, comparing the highest and lowest mercury quintiles). This association largely disappeared after adjustment for sociodemographic variables (income-poverty ratio, education, marital status). However, in age-stratified analyses, this inverse relationship remained in older adults (age ≥40) even after adjustment for sociodemographic variables. Simulation analyses adjusting for expected confounding effects of fish intake suggested that the inverse relationship among older adults may be plausibly attributed to residual confounding (Odds Ratio  = 0.75, 95% Confidence Interval: 0.50–1.12, comparing the highest and lowest mercury quintiles).

**Conclusions:**

Higher total blood mercury was not associated with increased odds of depression. The lower odds of depression in older adults with higher total blood mercury may be due to residual confounding.

## Introduction

Mercury in its various elemental and compound forms is a ubiquitous neurotoxin, with many different potential routes for human exposure, including air pollution, dental amalgams, fish consumption, and occupational settings.[1], [2] Exposure to high levels of mercury may increase the risk of psychiatric symptoms.[3], [4] For instance, residents of Minamata, Japan, an area with large-scale methylmercury pollution, exhibited increased levels of mood and behavioral dysfunction, compared to residents of a reference area.[5] Workers exposed to mercury at a fluorescent lamp plant scored higher on the Beck depression inventory compared to unexposed subjects.[6] Some emergency responders to the September 11^th^ attack developed symptoms of depression and anxiety, suspected to be attributable to the high levels of mercury and other heavy metal exposures as building materials were destroyed.[7]

While mercury is well-recognized as a neurotoxin, an association between high levels of mercury exposure and risk of depression does not necessarily mean that low levels of exposure carry a risk as well. Investigating possible health effects of low-level mercury exposure is important, especially given recent debate concerning regulations for mercury emissions in the U.S., and heightened public health awareness concerning mercury.

However, research on possible psychiatric effects of low-level mercury exposure has been limited. Animal models suggest that low levels of mercury may be associated with multiple psychiatric symptoms, including depressive symptoms.[8]–[10] For example, mice chronically exposed to methylmercury (0.5 mg/kg/day, a low dose for mice) during development showed depression-like behaviors, possibly mediated by the induction of oxidative stress.[10] Other animal models also suggest that the low-dose methylmercury toxicity may involve the disruption of the serotonergic system, a neuronal system heavily involved with depression.[11] Although the animal data regarding low-level mercury exposure and depression is suggestive, human studies have not yielded supporting evidence.

The Food and Drug Administration conducted an extensive literature review in 2009 regarding the potential health hazard related to dental mercury exposure.[12] Of the 34 studies reviewed, 2 human subject studies assessed depression: a retrospective cohort study (N = 20,000) in the New Zealand Defense Force that assessed mood disorders[12], [13] and a study among dentists and dental assistants that assessed depressive symptoms (N = 193).[12], [14] The studies found no association between dental amalgam or urinary mercury and depression, respectively (*P*>0.05).

Given the continuing public health concern regarding both low-level mercury exposure and depression, further observational studies of the general population are necessary. In the present study, we examine the cross-sectional associations of total blood mercury and depressive symptoms in a nationally representative sample of the US adult population.

## Materials and Methods

### Ethics statement

All NHANES studies are approved by the National Center for Health Statistics Research Ethics Review Board.[15] Survey participants are asked to provide written consent to allow information about their health to be collected for research purposes.[16], [17] All health information collected is strictly confidential and privacy is protected by public laws.[18]

### Study population

The NHANES is a survey conducted yearly by the Centers for Disease Control and Prevention. It is a major program of the National Center for Health Statistics[18] that combines interviews and physical examinations on a nationally representative sample of all ages across the country. NHANES has a complex, multistage, probability design that samples the non-institutionalized civilian US population. The sample does not include individuals in nursing homes, members of the armed force, US nationals living aboard, or institutionalized individuals.[19]

NHANES is comprised of 2 components: 1) a computer-assisted in-person interview administered to participants 16 years or older in which data are collected on demographics, socioeconomic status (SES), dietary behaviors, and other health-related questions (A proxy is used if individuals cannot answer the questions themselves.);[20] and 2) an examination that includes medical and dental examinations, physiological measurements and laboratory tests.[18] Each two-year survey cycle comprises a nationally representative sample.[19]

**Figure 1:**
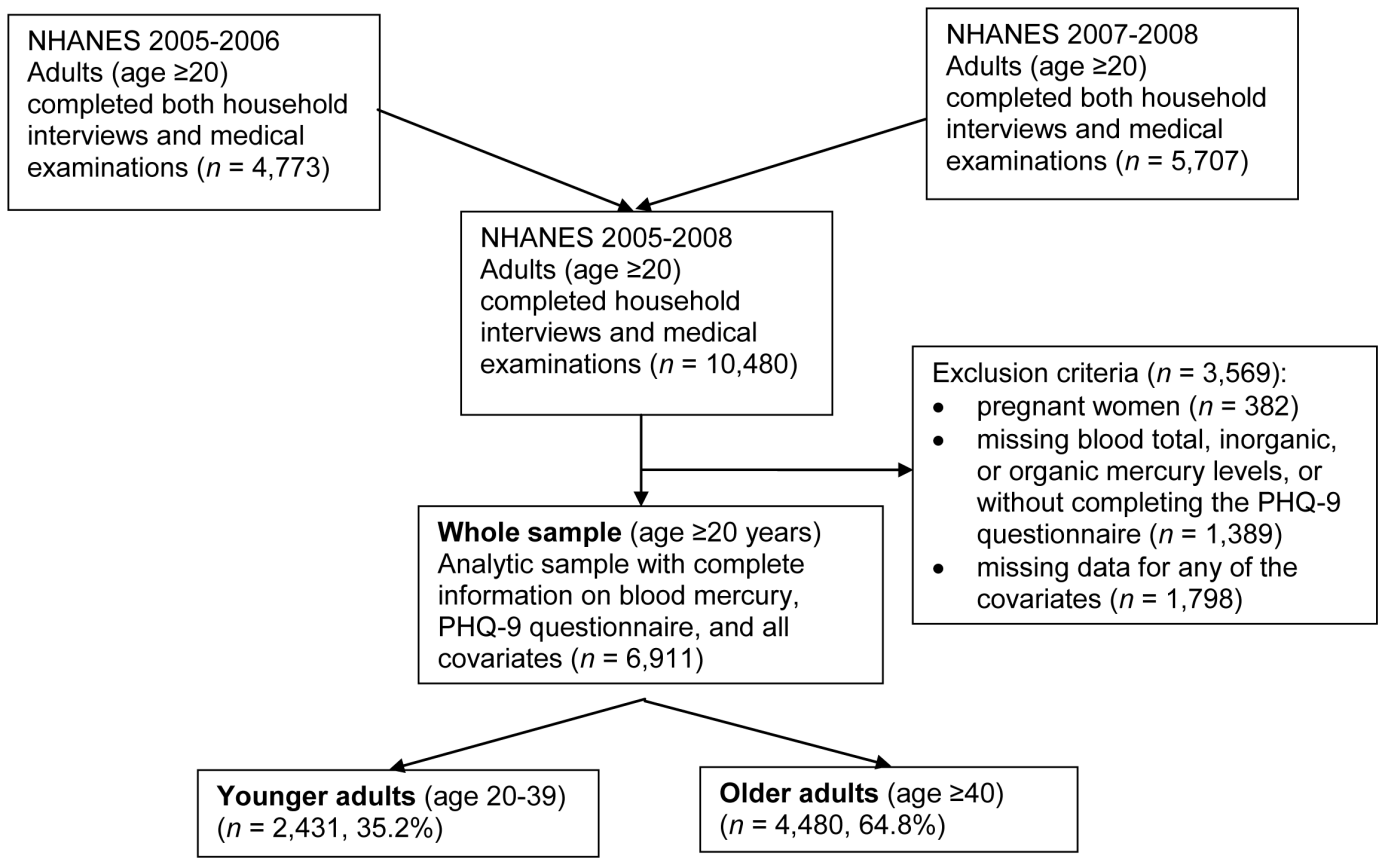
Study Population and Inclusion/Exclusion Criteria, NHANES, United States, 2005–2008. Abbreviations: NHANES–National Health and Nutrition Examination Survey; PHQ-9–Patient Health Questionnaire-9. Covariates included age, sex, race/ethnicity, family income-poverty ratio, educational attainment, marital status, current smoking status, self-reported drinking, body mass index, self-reported past 30 days fish intake, and past 24 hours polyunsaturated fatty acid intake. Percentages represented the percentages of individuals among the whole sample.

NHANES cycles 2005–2006 (*n* = 4,773) and 2007–2008 (*n* = 5,707) were used in this study. A total of 10,480 adults age ≥20 completed both household interviews and medical examinations (**Figure 1**). Pregnant women (*n* = 382) were excluded because pregnancy may modify the effects of mercury on depression.[2] Participants (*n* = 6,911) with complete data on important covariates (age, sex, race/ethnicity, family income-poverty ratio, educational attainment, marital status, current smoking status, self-reported drinking, body mass index (BMI), self-reported past 30 days fish intake, and past 24 hours polyunsaturated fatty acid (PUFA) intake were included in the analysis.

### Measurements

#### Depression

The Patient Health Questionnaire-9 (PHQ-9) was used to screen for depressive symptoms.[21] The instrument was administered through computer-assisted personal interviews at the NHANES mobile examination center.[21] The PHQ-9 has 9 items based on the 9 criteria of the *Diagnostic and Statistical Manual of Mental Disorders, fourth edition*, for diagnosing depressive disorders.[22], [23] Each of the 9 items scores from 0 (not at all) to 3 (nearly every day), resulting in a total of 0–27.[23] The PHQ-9 has been shown to be a reliable and valid indicator of depressive symptom severity.[23] The sensitivity and specificity of the instrument have been tested extensively in primary care and other clinical settings.[23] Scores between 0–4 generally indicate the absence of a depressive disorder.[23] Scores of ≥10 have a specificity and sensitivity of 88% for diagnosing with major depressive disorders.[23] In this study, individuals were categorized as having no depression (no depressive spectrum disorder; PHQ-9 score 0–4), or depression (high likelihood of a depressive spectrum disorder diagnosis; PHQ-9 score ≥5).

#### Laboratory methods

Blood samples were collected at the mobile examination center. Detailed blood sampling eligibility criteria and laboratory procedures are publicly available on the NHANES website.[24] In brief, total blood mercury was assessed by inductively coupled plasma mass spectrometry.[24] The limit of detection was 0.33 ug/L for 2005–06 and 0.28 ug/L for 2007–08. Because inorganic blood mercury levels were generally below the limit of detection (data not shown), the total blood mercury largely represents recent organic mercury exposure. The serum cotinine levels were measured by tandem mass spectrometry to assess the current smoking status of participants.[25]

#### Other covariates

A wide range of covariates were assessed in analysis. Variables included: age, sex, race/ethnicity, family income-poverty ratio (defined using US census methods[20] and guidelines from the Department of Health and Human Services for determining eligibility for federal programs, updated annually),[20], [26] educational attainment, marital status, current smoking status (assessed by serum cotinine level), self-reported drinking, and BMI (calculated based on measured height and weight). Fish consumption and fish oil, additional potential confounders for the association between mercury and depression,[27], [28] were assessed with dietary recall questions that included self-reported past 30 days fish intake and past 24 hours PUFA intake (docosahexaenoic acid (DHA) or eicosapentaenoic acid (EPA); intake values were calculated from the amount of food consumed 24 hours prior to the dietary recall day and the nutritional information provided by the United States Department of Agriculture).[29]

### Statistical analysis

Survey data from NHANES 2005–2006 and 2007–2008 were combined to form a single data set. Sample weights were adjusted according to NHANES guidelines to generate a nationally representative sample.[19] Total blood mercury was divided into quintiles for analysis. Cases of high likelihood of a depressive spectrum disorder diagnosis were identified as PHQ-9 scores ≥5. Age was categorized into 5-year intervals. Race/ethnicity included non-Hispanic white, non-Hispanic black, Mexican American, and other/multiracial. Income-poverty ratio was categorized into quintiles. Educational attainment was defined as individuals who: 1) did not complete high school, 2) completed high school or some college, and 3) completed college or above. Marital status included married individuals, individuals living with partner, widowed/divorced/separated individuals, and those who were never married. Current smokers were defined as serum cotinine >3 ng/mL;[30] otherwise, non-smokers. Self-reported drinking was categorized into two groups–individuals who, in the past year, consumed alcohol on average <1 day/week or ≥1 day per week. Individuals with BMI<18.5, 18.5 to <25.0, 25.0 to <30.0, and ≥30.0 were defined as underweight, normal, overweight, and obese, respectively.[31] Past 30 days fish intake was a dichotomous (yes/no) measure. Past 24 hours EPA and DHA intake were categorized into tertiles for analysis.

Because the association between blood mercury and depression may differ by age,[3] the analytic sample was stratified into younger adults (age 20–39) and older adults (age ≥40). Blood mercury levels, depression prevalence and covariate characteristics were compared between the age strata; depression prevalence was compared across blood mercury quintiles and age strata. Chi-square tests were used to identify any differences between these characteristics. Survey-weighted logistic regression models were used to examine the association between total blood mercury and depression: 1) Model 1: adjusted for age, sex and race/ethnicity; 2) Model 2: Model 1 covariates + income-poverty ratio, educational attainment and marital status; 3) Model 3: Model 2 covariates + current smoking status, self-reported drinking and BMI; 4) Model 4: Model 3 covariates + fish and PUFA intake.

Additional analyses were performed to examine the potential confounding effects of fish intake. Higher fish consumption has been shown to be associated with a lower risk of depressive symptoms.[32], [33] In NHANES, neither the self-reported past 30 days fish intake (yes/no) or the 24-hour dietary recall can accurately represent habitual fish consumption.[34] Exposure misclassification could plausibly lead to the attenuation of the fish intake and depression association (observed Spearman correlation *r* = −0.03; OR adjusted for all covariates  = 1.03), which is weaker than other studies have suggested.[32], [33] In order to examine the potential confounding of the mercury-depression relationship by fish consumption, random normal variables were generated to simulate fish intake. These variables had similar Spearman correlations with total blood mercury as the self-reported past 30 days fish intake variable (*r* = 0.39); however, based on the literature,[32], [33] we specified a range of plausible correlations with PHQ-9 scores that were stronger (*r* = ranging from −0.11 to −0.54). The simulation logistic regression model adjusted for covariates in model 4 and replaced the self-reported past 30 days fish intake with the simulated fish intake measure in tertiles.

All analyses were 2-tailed with a significance level of 0.05. SAS survey procedures were used to account for the complex survey design of NHANES. All analyses were performed using SAS 9.2 (SAS Institute Inc., Cary, NC) and R 2.15.2.

## Results

After exclusion criteria were applied, there were a total of 6,911 individuals in the whole sample (**Figure 1**). Demographic characteristics were compared between included and excluded individuals of age ≥20 (results not shown). Included and excluded individuals did not differ by sex (*P*>0.1), but they differed by age and race/ethnicity (*P*<0.0001). Excluded individuals were older and were more likely to be non-Hispanic white.

The analysis for the whole study sample showed that 20.8% had elevated depressive symptoms (Table 1). The prevalence of depression was not different between younger and older adults even though younger adults had a slightly higher prevalence for depression (21.6% vs 20.4%). Most of the study population (95.8%) had blood mercury below the Environmental Protection Agency reference dose of 5.8 µg/L. The median level of total blood mercury of the study population was 1.0 µg/L (interquartile range: 0.5–2.0 µg/L). Compared to younger adults, older adults had slightly higher total blood mercury levels (weighted median: 1.1 µg/L compared to 0.8 µg/L).

**Table 1:** Descriptive Statistics of the Study Population, NHANES, United States, 2005–2008.^a^

	Whole sample	Younger adults	Older adults
	N (%)	N (%)	N (%)
**Total**	6,911 (100)	2,431 (100)	4,480 (100)
**Depression**			
No (PHQ-9 score <5)	5,407 (79.2)	1,889 (78.4)	3,518 (79.6)
Yes (PHQ-9 score ≥5)	1,504 (20.8)	542 (21.6)	962 (20.4)
**Total blood mercury** b			
Quintile 1	1,481 (19.7)	490 (19.5)	995 (19.4)
Quintile 2	1,466 (20.1)	502 (19.5)	982 (20.5)
Quintile 3	1,418 (19.9)	529 (20.8)	870 (19.9)
Quintile 4	1,346 (20.2)	502 (20.1)	858 (20.0)
Quintile 5	1,200 (20.0)	408 (20.0)	775 (20.2)
Weighted median (IQR), µg/L	1.0 (0.5–2.0)	0.8 (0.5–1.7)	1.1 (0.6–2.1)
**Age**			
Weighted median (IQR)	45 (32–57)	29 (24–34)	54 (46–64)
**Sex** *			
Female	3,494 (52.4)	1,162 (48.4)	2,332 (54.9)
Male	3,417 (47.6)	1,269 (51.6)	2,148 (45.1)
**Race** *			
Non-Hispanic white	3,495 (73.6)	1,052 (66.1)	2,443 (78.3)
Non-Hispanic black	1,436 (10.5)	526 (11.8)	910 (9.7)
Mexican American	1,237 (7.6)	545 (11.5)	692 (5.1)
Other/Multiracial	743 (8.3)	308 (10.6)	435 (6.8)
**Family income-poverty ratio** b **^,^** c			
Quintile 1	2,011 (19.8)	654 (19.9)	1,402 (19.9)
Quintile 2	1,604 (20.2)	562 (20.1)	1,042 (20.1)
Quintile 3	1,270 (19.8)	496 (19.8)	774 (19.6)
Quintile 4	597 (11.1)	358 (19.7)	194 (6.0)
Quintile 5	1,429 (29.1)	361 (20.5)	1,068 (34.4)
Weighted median (IQR)	3.4 (1.7–5.0)	2.8 (1.4–4.6)	3.7 (1.9–5.0)
**Educational attainment** *			
Not completed high school	1,762 (16.2)	584 (17.0)	1,178 (15.7)
Completed high school or some college	3,648 (55.7)	1,365 (57.6)	2,283 (54.5)
Completed college or above	1,501 (28.1)	482 (25.3)	1,019 (29.8)
**Marital status** *			
Married	3,731 (56.2)	1,058 (44.0)	2,673 (63.7)
Living with partner	542 (8.1)	347 (13.9)	195 (4.6)
Widowed/divorced/separated	1,504 (18.7)	185 (7.4)	1,319 (25.7)
Never married	1,134 (17.0)	841 (34.7)	293 (6.0)
**Current smoking status** *			
Non-smoker (serum cotinine ≤3 ng/mL)	4,961 (71.3)	1,574 (63.7)	3,387 (76.0)
Current smoker (serum cotinine >3 ng/mL)	1,950 (28.7)	857 (36.3)	1,093 (24.0)
**Self-reported drinking**			
On average used alcohol <1 day/week in the past year	4,491 (60.4)	1,545 (58.7)	2,946 (61.5)
On average used alcohol ≥1 day/week in the past year	2,420 (39.6)	886 (41.3)	1,534 (38.5)
**Body mass index** *			
≥30.0 (Obese)	2,464 (33.7)	771 (29.5)	1,693 (36.3)
≥25.0–<30.0 (Overweight)	2,409 (33.9)	776 (30.2)	1,633 (36.1)
18.5–<25.0 (Normal)	1,934 (31.0)	840 (38.4)	1,094 (26.5)
<18.5 (Underweight)	104 (1.5)	44 (2.0)	60 (1.1)
**Self-reported past 30 days fish intake** *			
No	1,798 (23.6)	832 (31.0)	966 (19.0)
Yes	5,113 (76.4)	1,599 (69.0)	3,514 (81.0)
**Past 24 hours EPA intake** b			
Tertile 1	2,162 (32.0)	672 (28.5)	1,360 (30.7)
Tertile 2	2,366 (33.4)	917 (38.2)	1,663 (36.2)
Tertile 3	2,383 (34.5)	842(33.3)	1,457 (33.1)
Weighted median (IQR), gram	0.004 (0.001–0.017)	0.004 (0.000–0.017)	0.005 (0.000–0.017)
**Past 24 hours DHA intake** b			
Tertile 1	2,096 (31.6)	765 (32.8)	1,400 (32.3)
Tertile 2	2,469 (35.4)	783 (33.2)	1,592 (34.6)
Tertile 3	2,346 (33.1)	883 (33.9)	1,488 (33.1)
Weighted median (IQR), gram	0.022 (0.001–0.063)	0.020 (0.001–0.060)	0.024 (0.002–0.065)

Abbreviations: DHA–Docosahexaenoic Acid; EPA–Eicosapentaenoic Acid; IQR–interquartile range; NHANES–National Health and Nutrition Examination Survey; PHQ-9–Patient Health Questionnaire-9.

*P<0.05 for design-adjusted chi-square statistics based on weighted frequencies, comparing proportions between younger older adults.

a All proportions were calculated based on weighted frequencies; the frequencies presented here represented the sizes of the unweighted sample.

b Quantiles were generated based on the weighted sample. Chi-square tests were not performed for variables with quantiles. Quantile upper bounds (from left to right: quantile 1–5, respectively): Total blood mercury (µg/L)–whole sample: 0.5, 0.8, 1.3, 2.3, 38.7; younger adults: 0.4, 0.7, 1.1, 2.1, 23.8; older adults: 0.5, 0.9, 1.4, 2.5, 38.7; Family income-poverty ratio–whole sample: 1.4, 2.6, 4.0, <5.0, 5.0; younger adults: 1.2, 2.1, 3.5, <5.0, 5.0; older adults: 1.6, 2.9, 4.5, <5.0, 5.0; Past 24 hours EPA intake (gram)–whole sample: 0.001, 0.011, 2.589; younger adults: 0.001, 0.010, 2.589; older adults: 0.001, 0.012, 2.422; Past 24 hours DHA intake (gram)–whole sample: 0.005, 0.046, 6.390; younger adults: 0.004, 0.041, 6.390; older adults: 0.006, 0.047, 3.389.

c Note that the disproportional distribution of quintiles for income-poverty ratio was because all individuals in the 5^th^ quintile had the maximum value of 5.

Demographics, SES and other characteristics differed between younger and older adults (Table 1). Compared to the older adults, the younger adults consisted of more males, more ethnic minorities, fewer individuals who completed college or above, fewer married individuals, fewer current smokers, and a larger proportion with a lower BMI and lower income-poverty ratio. The younger adults also consumed more fish compared to the older population. The PUFA levels between the two age strata were similar.

For the whole sample analysis, the unadjusted logistic regression model suggested a negative association between total blood mercury and depression (Table 2, unadjusted model). The odds ratio (OR) for depression was 0.49, comparing the highest total blood mercury quintile to the lowest (95% confidence interval (CI): 0.36, 0.65). This association remained after adjusting for age, sex and race/ethnicity (Table 2, model 1). However, after additional adjustment for income-poverty ratio, educational attainment and marital status (model 2), the association between depression and total blood mercury disappeared, except for the highest quintile of total blood mercury. This largely null association persisted after further adjustment for current smoking status, self-reported drinking, and BMI (model 3), and disappeared after adjusting for past 30 days fish intake, and past 24 hours PUFA intake (model 4).

**Table 2:** Multivariate Logistic Regression Models for the Association Between Depression and Total Blood Mercury, Stratified by Age Group, NHANES, United States, 2005–2008.

Total blood mercury	Unadjusted		Model 1^a^		Model 2^b^		Model 3^c^		Model 4^d^		Simulation^e^	
	OR	95% CI	OR	95% CI	OR	95% CI	OR	95% CI	OR	95% CI	OR	95% CI
**Whole sample**												
Quintile 1	1.00		1.00		1.00		1.00		1.00		1.00	
Quintile 2	0.84	0.69, 1.04	0.84	0.68, 1.03	0.96	0.79, 1.16	0.96	0.79, 1.18	0.95	0.76, 1.18	1.00	0.82, 1.23
Quintile 3	0.74*	0.56, 0.98	0.74	0.55, 1.00	0.92	0.69, 1.23	0.94	0.70, 1.25	0.92	0.68, 1.25	0.98	0.73, 1.33
Quintile 4	0.62*	0.48, 0.79	0.61*	0.47, 0.80	0.78	0.60, 1.01	0.80	0.60, 1.05	0.78	0.59, 1.04	0.89	0.67, 1.17
Quintile 5	0.49*	0.36, 0.65	0.48*	0.36, 0.65	0.71*	0.54, 0.93	0.74*	0.56, 0.97	0.73	0.53, 1.00	0.98	0.70, 1.37
**Younger adults (20–39 years)**												
Quintile 1	1.00		1.00		1.00		1.00		1.00		1.00	
Quintile 2	1.14	0.80, 1.62	1.11	0.77, 1.62	1.21	0.83, 1.75	1.22	0.83, 1.80	1.19	0.80, 1.76	1.27	0.86, 1.87
Quintile 3	0.88	0.59, 1.30	0.85	0.55, 1.33	0.97	0.63, 1.51	0.99	0.62, 1.56	0.96	0.60, 1.54	1.04	0.65, 1.67
Quintile 4	0.89	0.55, 1.43	0.87	0.52, 1.48	1.07	0.62, 1.87	1.12	0.62, 2.01	1.09	0.62, 1.93	1.23	0.71, 2.15
Quintile 5	0.66	0.41, 1.08	0.64	0.38, 1.08	0.86	0.52, 1.43	0.89	0.52, 1.51	0.86	0.50, 1.50	1.10	0.62, 1.95
**Older adults (≥40 years)**												
Quintile 1	1.00		1.00		1.00		1.00		1.00		1.00	
Quintile 2	0.65*	0.50, 0.85	0.65*	0.50, 0.84	0.76*	0.59, 0.98	0.77*	0.60, 0.98	0.76*	0.60, 0.98	0.80	0.62, 1.04
Quintile 3	0.72	0.51, 1.01	0.73	0.52, 1.04	0.97	0.70, 1.37	1.00	0.72, 1.40	1.00	0.71, 1.39	1.05	0.74, 1.50
Quintile 4	0.47*	0.34, 0.64	0.47*	0.35, 0.64	0.63*	0.46, 0.85	0.64*	0.46, 0.88	0.63*	0.45, 0.88	0.71	0.50, 1.02
Quintile 5	0.37*	0.26, 0.51	0.37*	0.26, 0.52	0.56*	0.41, 0.77	0.58*	0.42, 0.81	0.58*	0.40, 0.83	0.75	0.50, 1.12

Abbreviations: CI–Confidence Interval; NHANES–National Health and Nutrition Examination Survey; OR–Odds Ratio.

*P<0.05.

a Model 1: adjusted for age in 5-year intervals, sex, and race/ethnicity.

b Model 2: adjusted for family income-poverty ratio in quintiles, educational attainment, and marital status in addition to covariates in model 1.

c Model 3: adjusted for current smoking status, self-reported drinking, and body mass index (BMI) in addition to covariates in model 2.

d Model 4: adjusted for self-reported past 30 days fish intake, and past 24 hours PUFA (EPA or DHA) intake in tertiles in addition to covariates in model 3.

e Simulation: adjusted for covariates in model 4, except that past 30 days fish intake (self-reported yes/no; *r* = −0.03 with depression) was replaced by simulated fish intake (normally distributed; *r* = −0.15 with depression) in tertiles; the simulated variable was generated based on the assumption that fish intake and depression were at least mildly correlated if recall bias to the self-report measure could be reduced.

In age stratified analyses, logistic regression models suggested that the associations between total blood mercury and depression differed by age (Table 2). Total blood mercury was not associated with depression among younger adults in both unadjusted and adjusted models.

In contrast, among older adults, total blood mercury was negatively associated with depression. After adjustment for income-poverty ratio, educational attainment and marital status (Table 2, model 2), OR estimates were attenuated, but the statistical significance of the ORs persisted. This negative association between total blood mercury and depression persisted after additional adjustment for other covariates (models 3 and 4).

A fish intake measure was simulated to further examine whether fish consumption may confound the inverse relationship between total blood mercury and depression among older adults. In models adjusting for the simulated fish intake instead of past 30 days fish intake, the highest tertile of simulated fish intake predicted a lower odds of depression, compared to the lowest tertile. For example, with the simulated variable specified at a mild correlation with PHQ-9 scores (*r* = −0.15), the OR of depression comparing the highest to the lowest tertile of fish intake was (OR = 0.58, 95% CI: 0.44, 0.78; data not shown). This relationship was in contrast to the null association between the self-reported past 30 days fish intake and depression in model 4 (OR = 1.03, 95% CI: 0.76, 1.38, comparing individuals who consumed fish in the past 30 days to those who did not; data not shown). In addition, the simulation logistic regression model showed that higher total blood mercury was no longer associated with increased risk for depression among older adults. With the simulated fish intake variable set at a stronger correlation with PHQ-9 scores (*r*≤−0.42), the relationship between mercury and depression became positive, suggesting that higher blood mercury was associated with higher risk of depression.

## Discussion

In this nationally representative sample, the overall mercury burden was low, with over 95% of persons having total blood mercury levels falling below the Environmental Protection Agency reference dose of 5.8 µg/L,[35], [36] an exposure without recognized adverse health effects. In unadjusted analyses, higher blood mercury levels appeared to be associated with reduced odds of depression among older but not younger adults. However, adjustment for family income-poverty ratio, educational attainment, and marital status lessened the extent of the inverse relationship between depression and blood mercury among older adults. This association persisted after further covariate adjustment, but disappeared after adjusting for simulated fish intake.

Mercury is a known neurotoxin that may lead to psychiatric symptoms possibly by exerting oxidative stress on the central nervous system, which might disrupt the metabolism of serotonin.[11] No empirical evidence suggests that mercury might protect against depression, and therefore our unadjusted results of low-level total blood mercury protecting against depression were unexpected. Our stratified, multivariate analyses suggested that the unadjusted association was largely, but not completely, confounded by income-poverty ratio, educational attainment, and marital status among older adults.

SES, measured by income-poverty ratio and educational attainment in this study, is a potentially important confounder of the blood mercury and depression relationship. Individuals with a lower SES, as well as individuals previously married, may have a higher risk for depression, possibly mediated by exposures to a wide range of stressors.[37] Compared to individuals with a lower SES, adults with a higher SES may consume more fish,[38], [39] thus exposing these individuals to more methylmercury. Because the total blood mercury in this study largely reflected organic mercury exposure, a positive relationship between SES and total blood mercury was plausible. The lower risk of depression among individuals with a higher SES may counter the toxic effects due to the increased exposure to methylmercury, thus making SES a potential negative confounder for the mercury-depression relationship. Lack of control for SES may lead to an underestimation of the toxicity of total blood mercury.

Our results support that SES was a negative confounder to the relationship between mercury and depression because the inverse mercury-depression association among older adults was reduced after controlling for SES. The persistent inverse association after SES adjustment may be due to incomplete control of potential SES covariates for older adults. The older adults in this study consisted of individuals who had been in the work force for a prolonged period of time and individuals of retirement age. It is possible that income did not adequately reflect the SES among older adults. In particular, retired individuals receiving pensions may have a similar monthly source of income to that of a worker of the same age. Accurate measurements of the accumulation of assets, such as savings and ownership of properties, are better SES indicators for these individuals compared to income.[40] Without such measures, misclassification of SES for older individuals may occur. The inability of income-poverty ratio to adequately reflect SES, as well as the limited availability of important SES measures in NHANES, for older adults may explain the persistent inverse relationship between mercury and depression. Proper controlling for confounding by SES is necessary to unveil the true association between low-level blood mercury and depression.

Fish intake is a potential confounder for the association between blood mercury and depression.[27] The relationship between fish consumption with health outcomes is complex because seafood is often contaminated by various pollutants, including mercury, while it is also a source of essential nutrients, such as PUFA. In particular, the literature suggests that two forms of PUFA, EPA and DHA, may protect against or treat depression.[28] Because of the dual relationship with health outcomes, fish intake may negatively confound the association between mercury and depression.[27] Adjustment for dietary intake was particularly important for this study because the blood mercury in the sample population was largely composed of organic mercury, of which fish is a major source. In this study, self-reported fish intake in the past 30 days and the amount of EPA and DHA consumed in the past 24 hours were used to control for the potential negative confounding effect. Although the inverse relationship between total blood mercury and depression persisted after adjustment for these variables, additional analyses adjusting for simulated fish intake found no or positive association between total blood mercury and depression.

Some evidence suggests that diets with high intake of fish may protect against depressive symptoms.[32], [33] However, the self-reported past 30 days fish intake measure in this study was very weakly related to depression. Due to the self-reported nature of dietary measures in NHANES, under-reporting was possible.[41] In addition, the PUFA levels were derived from self-reported food consumption in the past 24 hours. Serum measures of PUFA were not available to verify the accuracy of the food-derived measures. As well, the levels of methylmercury and PUFA vary depending on fish species.[42], [43] If biases due to self-report and lack of objective measurements can be overcome, dietary consumption may explain the inverse relationship between total blood mercury and depression. This speculation is supported by our simulation analysis which used a fish intake measure that predicted lower odds of depression by increased fish consumption, an association comparable to other studies.[32], [33] The unexpected mercury-depression relationship among older adults disappeared after adjusting for the simulated fish intake. Detailed information on individual food and nutrient intake is necessary to understand the true confounding effects from fish and PUFA.

It should be noted that additional confounders to the association between blood mercury and depression may exist. For instance, fish contains other nutrients, such as selenium, which may be associated with depressive symptoms.[27], [44] Accounting for additional potential confounders may help explain the inverse relationship between total blood mercury and depression.

This study has additional limitations. The cross-sectional design, and thus the lack of temporal information, did not permit the examination of the causal relationship between mercury and depression. In addition, the blood mercury levels in participants reflected their recent exposures to mercury (half-life of blood mercury: ∼50 days).[35], [45], [46] Therefore, the relationship of current health conditions with past exposure or accumulated levels of mercury is unclear. The PHQ-9 assessed current depression of participants based on self-reported symptoms in the past 2 weeks.[22], [47] Therefore, past or chronic depressive symptoms that may be associated with chronic, low-level mercury exposure may not be captured in this study. Further research with a prospective study design may help clarify the temporal relationship between mercury and depression.

In conclusion, this study found no evidence that the low-level blood mercury in the US adult population in 2005–2008 increased risk for depression. A reduced risk of depression among older adults with a higher total blood mercury was observed, but this potentially chance association may be explained by residual confounding due to measures such as SES and fish consumption. The strength of this study included the use of a large sample size that was nationally representative, the consideration for multiple SES factors at individual and household levels, the control for dietary intake, and the use of simulation to examine potential negative confounding effects. Further research with comprehensive adjustment for SES and serum measures for contaminants and nutrients from fish is needed to investigate the nature of the association between low-level blood mercury and depression.
